# Editorial: Community series in unveiling immunological mechanisms of periodontal diseases, volume II

**DOI:** 10.3389/fimmu.2025.1571093

**Published:** 2025-02-21

**Authors:** Pedro Paulo Chaves de Souza, Teun J. de Vries

**Affiliations:** ^1^ The Innovation in Biomaterials Laboratory, School of Dentistry, Federal University of Goiás, Goiânia, Brazil; ^2^ Department of Periodontology, Academic Centre for Dentistry Amsterdam (ACTA), Amsterdam, Netherlands

**Keywords:** periodontitis, Mendelian randomization, scRNAseq, inflammation, bone resorption

It remains a scientific challenge to study the immunological mechanisms of complex diseases associated with the inflamed periodontium, the tissues surrounding our teeth. Periodontitis, a chronic inflammatory disease that is associated with bone loss, is particularly difficult to investigate due to its alternating active and silent phases. The dysbiotic biofilm that is associated with the disease can vary between patients, disease stages and sample sites. Mouse models can help mechanistically but may have shortcomings in mimicking dysbiosis. Understanding the characteristics of patient cohorts may help to further characterize inflammation-related parameters.

The present series is the continuation of the previous series on “Unveiling Immunological Mechanisms of Periodontal Disease” (1). Compared to the previous series, the present one has shifted to themes that were not addressed three years ago, probably reflecting the novel tools that are available through knowledge of the genome and emerging techniques such as single-cell RNA sequencing (scRNA-seq). This Research Topic has contributed to the following themes.

## Cell biology models

Understanding the cellular and molecular mechanisms that drive periodontal inflammation and tissue destruction remains the basis of periodontal research. Advances in systems biology and cutting-edge transcriptomic technologies have enabled researchers to dissect the complex interactions between immune cells, stromal cells, and microbial communities in the periodontium. The following studies exemplify how cell biological models are elucidating the immunomodulatory roles of specific cell populations and pathways, offering new insights into the pathogenesis of periodontitis and potential therapeutic targets.

The study by Kim et al. revealed a pivotal role for ICAM1+ gingival fibroblasts as immunomodulatory sentinels in periodontal inflammation. Through integrative analysis of human scRNA-seq datasets, the authors demonstrated that ICAM1, the cell-cell adhesion molecule that fibroblasts can use to interact with immune cells, marks a fibroblast subset that expresses an inflammatory signature. This population orchestrates macrophage recruitment via CCL2, enabling efferocytosis to resolve neutrophilic inflammation, a process critical for mitigating tissue destruction. These findings redefine stromal-immune crosstalk in the periodontal niche and highlight ICAM1+ fibroblasts as a therapeutic target to modulate inflammation-driven bone loss.

Zhao et al. examined the role of the mechanosensitive ion channel Piezo1 in gingival destruction linked to periodontitis. Piezo1 expression is upregulated in the gingival tissue of periodontitis patients and drives macrophage polarization toward the M1 phenotype, leading to pro-inflammatory cytokine production and activation of MMPs, contributing to tissue destruction. The study suggests that inhibiting Piezo1 may reduce inflammation and collagen degradation, making it a potential therapeutic target for periodontitis.

Hu et al. investigated the role of miR-199a-5p in bone regeneration during apical periodontitis (AP), a disease marked by periapical inflammation and alveolar bone loss. Using transcriptomic analysis of clinical samples, the authors identified miR-199a-5p as significantly downregulated in AP tissues. Functional studies revealed that miR-199a-5p overexpression enhanced the proliferation and osteogenic differentiation of human stem cells from the apical papilla (hSCAPs), while its inhibition suppressed these processes. Mechanistically, miR-199a-5p targets *IFIT2*, a gene linked to type I interferon signaling, thereby alleviating its suppressive effects on osteogenesis. Furthermore, *in vivo* experiments demonstrated that hSCAPs overexpressing miR-199a-5p, when loaded onto β-tricalcium phosphate scaffolds, significantly enhanced ectopic bone formation in mice. These findings underscore miR-199a-5p as a critical regulator of bone repair in AP.

Taken together, these studies highlight the multifaceted roles of fibroblasts, mechanosensitive pathways, and miRNAs in periodontal inflammation and bone remodeling, offering new avenues for therapeutic intervention.

## Periodontal pathogens and periodontitis

Periodontitis can elicit antibody production against proteins of periodontal pathogens such as *Porphyromonis gingivalis*. A large, well-characterized cohort study such as the PerioGene North case-control study could determine whether antibodies against periodontal pathogens such as anti- arginine gingipains (Rgp), are associated with disease progression. Serum-Rgp IgG levels were clearly elevated in periodontitis patients compared to controls, and were even higher in patients with a high degree of inflammation and with alveolar bone loss Kindstedt et al.

To further explore the interactions of periodontal pathogens with the human immune system, Irwandi et al. proposed the use of the skin blister model to study the immunopathogenesis of periodontal disease. This model offers a controlled environment to explore localized host-pathogen interactions, bridging the gap between ex vivo studies and clinical observations, further advancing our understanding of the systemic links to periodontal inflammation.

## Mendelian randomization

Mendelian randomization is a relatively novel method that allows access to causal relationships between risk factors and health outcomes using genetic variants as instruments to infer causal effects (2). The current Research topic includes a study on the description of the role of tumor necrosis factor-receptor 1 TNF-α by Alayash et al., an article on interleukin-6 signaling by Nolde et al. and one on telomere length by Hu et al. While the first study did not find an association between TNF-receptor inhibition and periodontits., the interleukin study demonstrated that downregulation of IL-6 signaling based on genetic information was associated with lower odds of periodontitis. As we age, telomeres shorten (3). Since periodontitis increases with age, one would expect that shorter telomere length correlates with periodontal disease. Hu et al. showed indeed a reverse causal relationship, with shorter telomeres being linked to a higher risk of periodontitis, but no additional effect of telomere length and periodontitis when corrected for age.

## Reviews

Finally, this Research Topic also contains two state-of-the-art reviews. Novel research shows that chewing is beneficial to the aging periodontium and may help individuals maintain their teeth into old age (4). Mechanical forces can affect periodontal health through multiple mechanisms. Mechanical forces can influence soft and hard tissue metabolism. Excessive forces can damage the periodontium or result in irreversible inflammation. In their review, Wang et al., described the effect of mechanical forces on the parameters of the periodontium.

The review by Zhang et al. highlighted how emerging omics tools, such as RNAseq are revealing dynamic shifts in epithelial, stromal, and immune cell populations that drive inflammation and bone resorption, revolutionizing our understanding of immunopathology. However, as exemplified throughout this series, these discoveries require rigorous experimental validation. Integrating multi-omics approaches with mechanistic models and clinical cohorts will be critical to unravel the heterogeneity of dysbiotic biofilms and host responses. Together, this synergy of technologies and validation frameworks promises to advance precision therapies that address both the oral and systemic dimensions of periodontal disease.
